# Association of Long-Term Near-Highway Exposure to Ultrafine Particles with Cardiovascular Diseases, Diabetes and Hypertension

**DOI:** 10.3390/ijerph14050461

**Published:** 2017-04-26

**Authors:** Yu Li, Kevin J. Lane, Laura Corlin, Allison P. Patton, John L. Durant, Mohan Thanikachalam, Mark Woodin, Molin Wang, Doug Brugge

**Affiliations:** 1 Department of Rheumatology, Allergy and Immunology, Massachusetts General Hospital, Boston, MA 02114, USA; yul079@mail.harvard.edu; 2 Yale School of Forestry and Environmental Studies, Yale University, New Haven, CT 06511, USA; k.lanejr@gmail.com; 3 Department of Environmental Health, Boston University School of Public Health, Boston, MA 02118, USA; 4 Department of Civil and Environmental Engineering, Tufts University, Medford, MA 02155, USA; lauracorlin25@gmail.com (L.C.); allison.patton@alumni.tufts.edu (A.P.P.); john.durant@tufts.edu (J.L.D.); m_woodin@yahoo.com (M.W.); 5 Environmental and Occupational Health Sciences Institute, Rutgers University, Piscataway, NJ 08854, USA; 6 Department of Public Health and Community Medicine, Tufts University School of Medicine, Boston, MA 02111, USA; mohan.thanikachalam@tufts.edu; 7 Channing Division of Network Medicine, Brigham and Women’s Hospital and Harvard Medical School, Boston, MA 02115, USA; stmow@channing.harvard.edu; 8 Department of Epidemiology, Harvard T.H. Chan School of Public Health, Boston, MA 02115, USA; 9 Department of Biostatistics, Harvard T.H. Chan School of Public Health, Boston, MA 02115, USA; 10 Jonathan M. Tisch College of Civic Life, Tufts University, Medford, MA 02155, USA

**Keywords:** ultrafine particles, ischemic heart disease, diabetes, hypertension, time-activity adjusted particle number concentration

## Abstract

Ultrafine particle (UFP) concentrations are elevated near busy roadways, however, their effects on prevalence of cardiovascular diseases, diabetes, and hypertension are not well understood. To investigate these associations, data on demographics, diseases, medication use, and time of activities were collected by in-home surveys for 704 participants in three pairs of near-highway and urban background neighborhoods in and near Boston (MA, USA). Body mass index (BMI) was measured for a subset of 435 participants. Particle number concentration (PNC, a measure of UFP) was collected by mobile monitoring in each area. Intra-neighborhood spatial-temporal regression models (approximately 20 m resolution) were used to estimate hourly ambient PNC at the residences of participants. We used participant time activity information to adjust annual average residential PNC values and assign individualized time activity adjusted annual average PNC exposures (TAA-PNC). Using multivariate logistic regression models, we found an odds ratio (OR) of 1.35 (95% CI: 0.83, 2.22) of TAA-PNC with stroke and ischemic heart diseases (S/IHD), an OR of 1.14 (95% CI: 0.81, 1.62) with hypertension, and an OR of 0.71 (95% CI: 0.46, 1.10) for diabetes. A subset analysis controlling for BMI produced slightly stronger associations for S/IHD (OR = 1.61, 95% CI: 0.88, 2.92) and hypertension (OR = 1.28, 95% CI: 0.81, 2.02), and no association with diabetes (OR = 1.09, 95% CI = 0.61, 1.96). Further research is needed with larger sample sizes and longitudinal follow-up.

## 1. Introduction

Exposure to airborne particles, especially particles <2.5 microns in aerodynamic diameter (PM_2.5_), has been shown to be associated with cardiovascular morbidity and mortality [1,2,3,4]. Ultrafine particles (UFP), airborne particles with an aerodynamic diameter <0.1 micron [5], could also plausibly affect cardiovascular health due to their small size and large surface area [6]. Nevertheless, despite evidence regarding associations of PM_2.5_ with hypertension [7,8] and diabetes [9,10,11], a limited number of studies have investigated the long-term effects of UFP on cardiovascular risks and clinical outcomes. A longitudinal study recently found an association between long-term exposure to neighborhood-scale (1 × 1 km resolution) UFP exposure and inflammatory and coagulation markers [12]. Urban scale (4 × 4 km resolution) UFP exposure was also recently reported to be associated with cardiovascular mortality [13]. A multi-city longitudinal study found significant associations between long-term exposure to particle number concentration (PNC) and carotid intima-media thickness, a subclinical marker for atherosclerosis [14]. Nevertheless, the spatial scales of the air quality models used in these studies did not account for within-neighborhood differences in UFP concentration. Unlike PM_2.5_, a regulated and more widely studied component of PM, UFP concentrations vary substantially in urban areas, and are particularly high within about 200 m of busy roads [15,16,17,18].

Toxicological evidence suggests that particulate air pollution may lead to cardiovascular morbidity through inflammation in the respiratory or circulatory systems, systemic oxidative stress, or via activation of the autonomic nervous system in the lungs [1]. A growing number of toxicologic experiments also suggest an association between PM_2.5_ and diabetes through an inflammatory pathway leading to endothelial dysfunction and increased insulin resistance, a major underlying abnormality driving diabetes [19,20,21,22]. Based on this evidence, we hypothesized that high UFP exposure near busy roadways may be associated with cardiovascular disease and its risk factors.

The Community Assessment of Freeway Exposure and Health Study (CAFEH) is a community-based participatory, cross-sectional investigation of the relationship between UFP and cardiovascular risks with fine-grain spatial (~20 m) and temporal (1 h) resolution in exposure assignment [23]. In a prior analysis of CAFEH data, we found non-significant positive associations between time-activity adjusted annual average PNC exposure (TAA-PNC) and C-reactive protein (hsCRP), interleukin-6 and tumor-necrosis factor alpha receptor II. We also found non-significant negative association with fibrinogen [24,25]. In this paper, we used the same high resolution, long-term exposure to TAA-PNC to develop explorative data on the prevalence of S/IHD (stroke, myocardial infarction, congestive heart failure, and/or angina), diabetes, and hypertension.

## 2. Materials and Methods

### 2.1. Study Population

**Figure 1:**
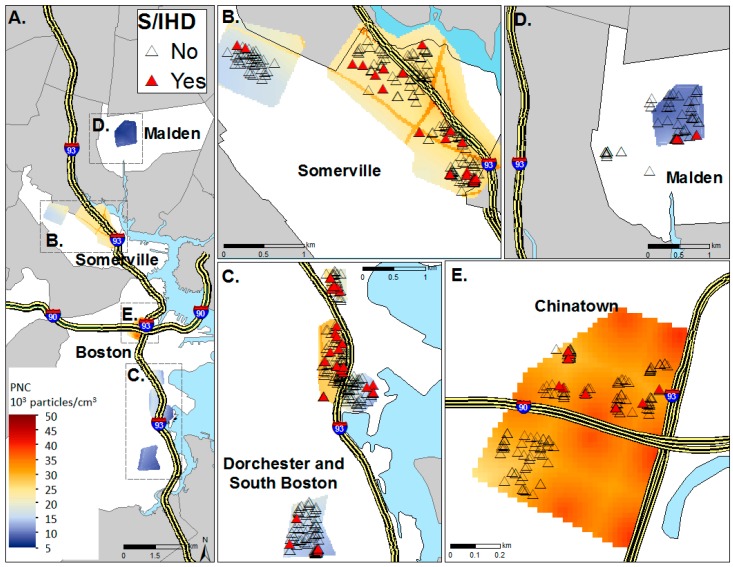
CAFEH study populations by status of stroke and ischemic heart diseases (S/IHD) in the neighborhoods of Somerville, Dorchester/South Boston, and Chinatown/Malden. (**A**) All study areas with annual average TAA-PNC levels; (**B**) The Somerville near highway and urban background study areas with participants by stroke/ischemic heart disease status (S/IHD); (**C**) Dorchester and South Boston near highway and urban background study areas with participants by S/IHD status; (**D**) The Malden (urban background for Chinatown) study area with participants by S/IHD status; (**E**) The Chinatown near highway study area with participants by S/IHD status.

Details of the design and methodology of the CAFEH study have been previously published [23] (see Appendix A) and are briefly described here. Data were collected from 704 participants, of whom 574 were randomly sampled and 129 were a convenience sample. A subset of 435 participants attended a field clinic. Participants were over 40 years old and from three pairs of near-highway and urban background communities in and near Boston (MA, USA). In order to increase exposure contrast, participants were recruited based on their residential proximity to Interstate Highway 90 (I-90) or 93 (I-93), with paired near-highway (≤500 m) and urban background (>1000 m) neighborhoods. Neighborhoods were matched demographically on race/ethnicity and socioeconomic status as much as possible. The study neighborhoods were in Somerville, Dorchester/South Boston, Boston Chinatown, and Malden (Figure 1). The study was approved by the Tufts Health Sciences Campus Institutional Review Board and all participants provided written informed consent.

### 2.2. Data Collection

The CAFEH study participants were recruited from 2009 to 2012 and completed an in-home survey that included demographics, smoking history, perceived stress scale (PSS), time-activity, medication use, self-report of diagnosis of health conditions, and other variables [23]. The questionnaires were conducted in each participant’s home by field surveyors in six languages in order to include linguistic minority populations. Medications were transcribed by investigators from medication containers in the home. Participants who had self-reported health outcomes were asked the number of years since diagnosis, measured as <1, 1–4, 5–9, 10–20, or >20 years because precise memory was assumed to be unreliable. We recorded the number of years and months living at the current address as a continuous variable. Participants at the field clinic provided weight and height that were used to calculate body mass index (BMI).

### 2.3. Exposure Assessment

We conducted a cross-sectional study that used each participant’s time-activity data to assign a single individualized annual average exposure to time-activity adjusted average particle number concentration (TAA-PNC). The methods of the TAA-PNC exposure assignment have been published in detail elsewhere [24], and a brief summary has been provided in the Appendix A. UFP concentrations were measured using a mobile laboratory [17,18] on 35 to 47 days in each neighborhood during the same year as participant data collection. The mobile laboratory was driven over fixed routes in each study area at different times of the day, days of the week, and seasons of the year for 12 months. Both near-highway and urban background air pollution concentrations were measured. UFP was measured as PNC (>4 nm in aerodynamic diameter) using a condensation particle counter (TSI CPC 3775, Shoreview, MN, USA). Hourly spatial-temporal regression models of the natural log of PNC (ln PNC) were developed with variables related to location, traffic, and meteorology [26,27]. We assumed that these exposure estimates would be relatively stable over 7–11 years, the time frame in which chronic diseases, including CVD, diabetes and hypertension, might be expected to develop in response to air pollution exposure. We based this assumption on studies showing stability of spatial patterns of other traffic-related air pollutants over these time periods [28,29,30]. This, in turn, makes sense since urban transportation systems, which are primarily responsible for PNC, change slowly over time. Participant addresses were verified by field staff during home visits and were geocoded by parcel and street network with manual correction to reduce positional error [31].

Our approach to adjusting exposure for participant time-activity patterns and assigning each participant with a cross-sectional measure of TAA-PNC exposure level has been published in detail elsewhere [24] (see the Appendix A). Briefly, ambient PNC was estimated for every hour of the year for each participant using neighborhood-specific regression models. PNC for each participant was calculated as the annual mean of the hourly assigned PNC values, adjusting for individual time-activity data for five micro-environments (inside home, outside home, at work, on highway, and other). PN exposures inside and outside homes were assigned as the residential ambient hourly average value, assuming equal indoor and outdoor PNC based on a previous analysis of PNC indoor/outdoor ratios for a subset of homes in our study population [32]. In addition, questionnaire data on seasonal air conditioner (AC) use was combined with temperature from local weather stations to adjust PNC inside homes. We assumed that AC use occurred when ambient temperatures exceeded 21 °C (70 °F) and during these times, we reduced exposure estimates by 25% for window AC units and 28% for central AC [32].

For participants who worked at jobs with traffic-related air pollution (TRAP) exposure (e.g., bus drivers, crossing-guards, or parking ticket officers), we assigned the mean hourly residential PNC of all participants residing ≤50 m from I-90 or I-93 for the hours they were at work. Participants without significant TRAP exposure at work (e.g., nurses, administrators, or school teachers) were assigned the average hourly residential PNC of all participants residing in the urban background area (≥1000 m from I-90 and I-93). For all other times (labeled “other”) that were not captured by the five primary categories (about 5% of time), we also assigned the hourly residential average of all participants residing in the urban background area since it was the most likely exposure. PNC exposures for time spent on state and interstate highways were assigned as the on-highway (I-93) concentration predicted by the Somerville PNC model [24]. We integrated all time-activity information and constructed one cross-sectional, annual average TAA-PNC level for each participant.

### 2.4. Outcome Assessment

The three health outcomes were the prevalence of S/IHD, diabetes, and hypertension. S/IHD was defined as self-reported doctor diagnosis of stroke, myocardial infarction, congestive heart failure, and/or angina. Diabetes and hypertension were defined as either reporting that diagnosis and/or taking relevant medications as determined by two physicians. The time since diagnosis was estimated using the mean value of the time range reported by each participant. This time was then compared to the time living at their current address to reduce the chance that outcomes preceded residence near the highways. Only cases with an estimated time of diagnosis after the time of moving to the current address were included (n = 57 for S/IHD, n = 73 for diabetes, n = 193 for hypertension). Since participants who were diagnosed with each disease before moving to their current address were only excluded from the corresponding health outcome, the number of participants analyzed varied by health outcome.

### 2.5. Statistical Methods

Potential confounders, selected a priori, included age, sex, born in the U.S., race, education, income (Pearson’s correlation between education and income = 0.28), smoking status, time of residence in current address, Perceived Stress Scale, work status, marital status, random or convenience sample, and length of time per week of light or moderate leisure-time physical activities. BMI was included in the analysis of the subset of participants for whom height and weight data were collected (n = 435). Community is defined in this study as a categorical variable that grouped participants based on study area and whether they resided in the near-highway or urban background areas. Community was not considered as a potential confounder due to its high collinearity with exposure and race. Race and ethnicity were heterogeneous due to small sample sizes in many sub-categories, thus we categorized the race variable into non-Hispanic white, Asian (East and South Asians), and other. Due to high collinearity between born in the U.S. and race, born in the U.S. was not entered in the main models. Instead, sensitivity analyses were performed substituting born in the U.S. for race in each fully-adjusted multivariate model. Participants with missing income data (n = 70) were categorized separately for analysis.

Logistic regression models were used to examine the association of mean TAA-PNC with S/IHD, diabetes, and hypertension. The first multivariate model was adjusted for sex, education (higher versus lower than high school diploma), and age. Binary education was used due to the limited number of cases of S/IHD and diabetes. A quadratic term for age was included in all models, in addition to the linear term, in order to address potential non-linear associations between age and health outcomes. We generated the second multivariate model using a forward stepwise selection, with the selection criteria of *p* < 0.20 to enter the model and of *p* ≥ 0.10 to be removed from the model. Using the same criteria, we included other major covariates that contributed to the first multivariate model (born in the U.S., race, income, smoking, work status, marital status, random or convenience sample, community, time of residence in current address, Perceived Stress Scale, and length of exercise per week). Effect size and standard error were assessed for each variable included in the model.

Potential effect modifiers examined were race (a variable found to be important in prior analyses of this study population: [25,33]), age (equal to or older versus younger than 60, the mean age), sex (M/F), random versus convenience sample, and smoking status. Additionally, a sensitivity analysis was performed using a stricter exclusion criterion that defined time of diagnosis as the earliest possible time in the time range of diagnosis. We then excluded all cases that had diagnosis times before the time of moving to the current address (in contrast to using the mean of the time range of diagnosis in the main analysis). A second sensitivity analysis was performed that additionally adjusted for diabetes and hypertension in a third multivariate model for S/IHD.

Since BMI is a known risk factor for all three health outcomes and the data are only available for the participants with clinical data, a subset analysis was performed that included BMI in the models. The same model building approach was used as for the larger sample. The second multivariate model using the forward stepwise selection was limited to diabetes and hypertension as dependent variables, due to the limited number of cases of S/IHD (n = 29) in the subset. Analyses were performed using SPSS 19 (SPSS, Chicago, IL, USA) and SAS 9.4 (SAS Institute Inc., Cary, NC, USA). The figure was created using ArcMap 10.4 (ESRI; Redlands, CA, USA).

## 3. Results

The sample consisted of 704 participants, of which 58% were female and the average age was 60 years (Table 1). There were 57 cases of S/IHD, including 14 with diagnosis of stroke, 26 with myocardial infarction, 19 with congestive heart failure, and 20 with angina. There were 73 cases of diabetes and 193 cases of hypertension. The mean ages of participants with S/IHD, diabetes, and hypertension were higher than those without. A larger proportion of participants reporting S/IHD diagnosis were born in the U.S. (61% vs. 45% for non-S/IHD), slightly lower than in a previous report (73%) from a subset of the study population analyzed here [33]. People with any of the outcomes were more likely to be in the convenience sample and reported less light to moderate exercise. Time of residence at current address was longer among people with each of the health outcomes, likely due to the exclusion of cases with diagnosis before moving to current address. Estimated time since diagnosis was similar for S/IHD and hypertension, but shorter for diabetes. Additionally, the proportion of participants having an estimated time since diagnosis exceeding 10 years was only 20% (n = 12) for S/IHD, 11% (n = 8) for diabetes and 15% (n = 29) for hypertension. 

For the subset of the population with BMI data available, the BMI levels of participants with S/IHD, diabetes, and hypertension were higher than those without. The distribution of TAA-PNC levels for the overall population, as well as for participants with and without each health outcome, are shown in Table 2. Although we observed a large spread in annual average TAA-PNC exposures in the study population (9.0 × 10^3^ to 35 × 10^3^ particles/cm^3^), with the average for the total population being 21 × 10^3^ particles/cm^3^, differences in PNC exposure levels between participants with and without disease outcomes were close to the measurement uncertainty of PNC (~1.0 × 10^3^ particles/cm^3^).

The results of the multivariate logistic regression analyses are presented in Table 3. In the second multivariate model, the OR of TAA-PNC with S/IHD was 1.35 (95% CI: 0.83, 2.22), an OR of 1.14 (95% CI: 0.81, 1.62) was observed with hypertension, and an OR of 0.71 (95% CI: 0.46, 1.10) for diabetes. Potential effect modifiers were tested by adding an interaction term with TAA-PNC in the second multivariate model. Asian race significantly modified the association between TAA-PNC and diabetes (*p* for interaction = 0.02). In subsequent stratified analysis, Asian and non-Asian participants demonstrated odds ratios of 1.53 (95% CI: 0.58, 4.02) and 0.48 (95% CI: 0.27, 0.88), respectively. Due to the small sample size of Asian with diabetes, the test for effect modification between East and South Asian was not performed. No other putative effect modifiers showed significant modification of the association between TAA-PNC exposure and any of the health outcomes.

We substituted born in the US for race in the second multivariate model and found associations consistent with using race for all three health outcomes. A sensitivity analysis adjusting for diabetes in the second multivariate S/IHD model showed a weak non-significant association (OR = 1.17, 95% CI: 0.69, 1.96) that was consistent with the main analysis. Nevertheless, additionally adjusting for hypertension resulted in loss of the association between TAA-PNC exposure and S/IHD (OR = 1.08, CI: 0.63, 1.84). Sensitivity analyses with a stricter definition of cases that excluded participants with a diagnosis time prior to moving to current address for the three health outcomes had similar numbers of cases (n = 46 for S/IHD, n = 62 for diabetes, and n = 161 for hypertension), and their associations with TAA-PNC exposure were consistent with the main analysis.

The results of the subset analysis for participants with BMI data (n = 435) are presented in Table 4. BMI was statistically significant in all of the models. A positive, non-significant association was observed for S/IHD in the partially-adjusted model (OR = 1.61, 95% CI: 0.88, 2.92) and for hypertension in the fully-adjusted model (OR = 1.28, 95% CI: 0.81, 2.02). In contrast to the negative association seen in the total population, PNC was not associated with diabetes in the model adjusted for BMI (OR = 1.09, 95% CI: 0.61, 1.96).

## 4. Discussion

This analysis explored the association of high resolution, individualized PNC exposure accounting for time-activity with self-reported S/IHD, diabetes, and hypertension. In this community-based study, associations of long-term exposure to TAA-PNC with self-reported diagnoses of S/IHD and hypertension had large confidence intervals and non-significant associations. These associations were consistent after controlling for BMI in a subset of the study population. In contrast, a possible negative association with self-reported diabetes was not observed after adjusting for BMI.

Three longitudinal studies have reported associations of long-term exposure to PNC with cardiovascular risk factors and mortality and reported findings that are broadly consistent with our analysis [12,13,14]. Ostro et al. [13] found significant associations of UFP and their chemical constituents with death from S/IHD as well as total cardiovascular mortality when exposure was assessed by a 4 × 4-km grid (over the state of California). Viehmann et al. [12] studied blood inflammatory and coagulation markers in a cohort study using 1km grid assignment of PNC exposure. They observed a borderline significant association of PNC with fibrinogen and a non-significant association with hsCRP. While these studies showed overall consistent results with our observation, neither assessed fine grain exposure to near-roadway UFP. The third study, Aguilera et al. [14], observed an association of long-term exposure to UFP, on a grid of 200 × 200 m, with subclinical atherosclerosis, measured by carotid intima-media thickness.

A prior analysis of data from the CAFEH study used a subset of the population in the analyses presented here that had complete data on all variables in the models and viable blood biomarkers (n = 408) [25]. With BMI in the model, associations were observed between TAA-PNC and biomarkers of inflammation and coagulation that were very similar to the association in our subset analysis that also included BMI [25]. These biomarkers are known to be correlated with S/IHD [34], correlations that were also observed in the CAFEH population (*p* = 0.05 for hsCRP with S/IHD). It is important to acknowledge differences between blood biomarkers and the disease variables analyzed here. Self-reported diagnosis of disease was confirmed by medications for two of our outcomes, diabetes and hypertension, and while this is not an entirely objective measure, is common practice for epidemiology. These diagnoses are likely more stable measures than subclinical biomarkers which are affected by short-term variation.

An association of UFP with cardiovascular health is plausible based on biological mechanisms. There are three hypothesized biological pathways: (1) systemic inflammation originating in the lungs; (2) particles/particle components entering the circulation; and (3) interaction with the autonomic nervous system [1]. Due to their small size, UFP can be inhaled deeply into the lungs and reach deep vascular spaces. They can also cross biological barriers and have a large surface area capable of carrying toxic molecules [35]. Plausibility is improved because studies have shown associations of other forms of PM (primarily PM_2.5_) with S/IHD and inflammatory markers. There is also evidence that short-term UFP exposure is associated with blood pressure, systemic inflammation, and coagulation biomarkers [36,37,38,39].

We did not observe associations of TAA-PNC with diabetes in our population. It has been postulated that PM could contribute to diabetes through various pathways including immune activation, the adipose system, hepatic effects and acting on the central nerve system [9,10,11,19,20,21,22]. Nevertheless, there are very few studies testing associations between UFP and diabetes. One epidemiology study reported a short-term association between the pre-diabetic marker HbA1c and indoor but not outdoor PNC exposure [40]. We observed effect modification by Asian race for the association between PNC exposure and diabetes, suggesting that being Asian may be associated with higher prevalence of diabetes in individuals with high PNC exposure. Since the non-Asian subgroup is quite heterogeneous, there could be further differences among other racial/ethnic subpopulations for which we did not have sufficient power to analyze due to small numbers of cases. Similarly, East and South Asian may have heterogeneity regarding diabetes and exposure to PNC; however, we were not powered to test the difference within Asian ethnic groups. We have previously reported that the Asian subpopulation in the CAFEH study differs from the white and other racial/ethnic subpopulations. The Chinese, who were most of the Asian population in this study, had lower blood biomarkers of inflammation and similar prevalence of diabetes and hypertension compared to U.S. born white participants [33]. Our previous analysis also reported statistically significant associations between PNC and inflammatory blood biomarkers in white, but not Asian participants [25].

In addition, we observed a non-significant association between PNC exposure and hypertension in the BMI-adjusted subset analysis. This is consistent both with previous studies showing significant association between PM_2.5_ exposure and hypertension in both cross-sectional and cohort studies [7,8] and a prior subset analysis on short-term effects in CAFEH [36].

Sensitivity of the results to variable selection and definition, and to inclusion criteria, was tested by repeating the analyses with different subsets of the population. Consistent associations were observed when substituting born in the U.S. for race in the fully-adjusted multivariate models. This makes sense as only a small proportion (37 of 314, 12%) of the white population was born outside of the U.S. while all of the Asians were first generation immigrants. In addition, for each health outcome we excluded all cases diagnosed prior to moving to their current address. To address the possibility that we included some individuals who were diagnosed before moving to their current address, a sensitivity analysis was performed using the longest possible time since diagnosis as a stricter exclusion criterion. We observed a small reduction of the number of cases but associations with PNC remained consistent with the main analysis. The sensitivity analysis adding hypertension as a covariate attenuated the association between PNC and S/IHD. One possibility is that hypertension may be on the causal pathway between PNC exposure and cardiovascular outcomes.

For exposure assessment, we utilized annual average PNC, a common practice for air pollution epidemiology [12,13,14]; we further strengthened our measurement by adjusting for participant time activity. PM_2.5_ exposure was not accounted for in our analysis, because measured concentrations of PM_2.5_ were essentially the same across the study area and were only weakly associated with PNC [18]. PM_10_ is composed of mainly non-tailpipe emissions [41], and was reported by studies to be less likely initiate an inflammatory response [42,43]. While PM_10_ from non-tailpipe emissions could confound the association between PNC and health outcomes, we did not measure PM_10_ in our study and could not account for its possible influence.

Our analysis has several strengths. First, TAA-PNC is based on high resolution models which are individualized for exposure based on participant time activity. This should reduce exposure misclassification, although it is possible that time activity patterns changed following diagnosis, something we could not assess. Our measures for hypertension and diabetes were strengthened by using medication as a criterion for assigning disease. We were also able to exclude most people who developed their illness prior to moving to the address at which we assigned exposure to PNC. Finally, as a community-based study with most participants randomly chosen and recruitment in six languages, there should be a degree of generalizability to older adults living in other urban near highway populations in the Northeastern U.S.

This study also has some limitations. We had a limited number of cases of S/IHD (n = 57) in the main analysis and an even smaller number (n = 29) in the sub-analysis controlling for BMI. This is because the CAFEH study was powered for blood biomarkers and not for chronic disease outcomes. This small number of cases weakened the stability of the adjusted logistic models. For future studies to detect a similar effect size with an expected power of 0.8, based on the association observed between S/IHD and PNC exposure in our study, we calculate that a minimum of 1128 participants would be needed [44]. There is also possible misclassification of self-reported disease status. We have reduced the misclassification in hypertension and diabetes by combining self-reported condition with medication. For S/IHD, because multiple mechanisms could lead to the development of congestive heart failure and stroke, there was the potential for misclassification. Also, due to the self-reported health outcomes in our study, we were not able to determine if strokes were ischemic or haemorrhagic. We therefore performed a sensitivity analysis excluding stroke and congestive heart failure, which resulted in a similar trend as in the multivariate adjusted model.

Not all the participants in our study had measured BMI, a key potential confounder in other work with this population [25], but we addressed this with analyses of the subset of our population that had BMI data. In addition, we were not able to assess the possible effects of other chemical and non-chemical exposures, including noise, which might confound associations with PNC. Also, including education in our models might not adequately control for SES.

We conducted indoor/outdoor monitoring at 18 homes in one of our study areas [32] and at 24 other homes in the Boston area (unpublished). These measurements suggest that ambient PNC is the major contributor to indoor PNC levels in our study population, and that indoor sources are a minor contributor. However, studies in other populations have shown that both indoor and outdoor activities can be contributors to indoor PNC concentrations [45,46]. Studies have also shown elevated indoor PNC levels in association with various indoor activities, including cooking [45]. Our assumption was that exposure to PNC was primarily from ambient sources. Accordingly, we may not have fully accounted for the total indoor levels of PNC. In addition, since PNC from indoor sources is also of different chemical composition than ambient sources [47], the indoor source contributions to PNC might also have different toxicity [45].

Despite our individualized exposure assessment that considered time activity of participants, there likely remains residual exposure misclassification in home and work micro-environments. Future studies monitoring more detailed, individualized level of monitoring that account for both indoor and outdoor micro-environments would have improved accuracy in determining the level of exposure of individual participants. Exposure misclassification relative to historical exposure from using present day exposure metrics could also have introduced error. We used time activity data collected during the cross-sectional study period to approximate the exposure levels, which may introduce exposure misclassification since the time-activity patterns of participants may change over time. The amount of bias in the TAA-PNC exposure assignment could potentially be greater in those participants with the S/IHD due to changes in time activity after diagnosis which we were not able to account for in our study design. While relative UFP levels likely remain stable over many years, the absolute levels might have a temporal trend. If relative levels remain similar, the main issue is likely to be the magnitude of ORs rather than their direction. Nevertheless, there is evidence that air pollution models may be relatively stable over a decade in fully developed cities where road networks, industrial emissions, and airports do not change [30]. Thus, for a study population having on average having an estimated time to diagnosis within four to six years this may be a reasonable assumption (Table 1).

## 5. Conclusions

Using high resolution and individualized TAA-PNC exposure levels to explore the problem of PNC spatial and temporal variation led to non-significant associations with S/IHD, hypertension and diabetes. There is a need for larger studies with longitudinal follow-up that use similar exposure metrics to ours to better assess whether near highway UFP are a risk factor for developing cardiovascular disease.

## Figures and Tables

**Table 1:** Demographic characteristics of total study population by health outcomes.

Participant Characteristics	Total	S/IHD ^1^		Diabetes ^2^		Hypertension ^3^	
	(n = 704)	Yes (n = 57)	No (n = 618)	Yes (n = 73)	No (n = 580)	Yes (n = 193)	No (n = 384)
**Female**	58%	56%	58%	58%	57%	63%	55%
**Age (years), mean (SD)**	60.0 (12.8)	67.8 (12.0)	59.0 (12.6)	64.9 (11.0)	59.0 (12.9)	66.0 (12.4)	55.3 (11.2)
**BMI (kg/m^2^), mean (SD) ^4^**	27.5 (6.8)	29.8 (7.0)	27.1 (6.6)	30.8 (7.2)	26.6 (6.00)	28.9 (6.9)	26.1 (5.99)
**Born in the U.S.**	46%	61%	45%	53%	46%	53%	43%
**Education**							
Less than high school diploma	33%	30%	33%	34%	32%	34%	28%
High school diploma	30%	28%	30%	41%	27%	34%	28%
Undergraduate college	26%	30%	26%	18%	29%	25%	30%
Graduate school	11%	12%	11%	7%	12%	7%	14%
**Race**							
White	46%	53%	45%	45%	47%	51%	47%
Asian	33%	25%	35%	26%	35%	31%	34%
Other	21%	23%	20%	29%	18%	18%	18%
**Income**							
$0–24,999	50%	56%	48%	63%	46%	56%	39%
$25,000–74,999	25%	32%	25%	16%	27%	23%	31%
$75,000 and more	15%	4%	16%	12%	16%	11%	20%
Don’t know/refused	10%	9%	10%	8%	10%	10%	11%
**Smoker status**							
Never	47%	37%	49%	34%	49%	42%	50%
Former smoker	29%	40%	28%	45%	28%	38%	26%
Current smoker	20%	21%	19%	16%	19%	17%	20%
Missing	4%	2%	4%	4%	4%	3%	4%
**Work status**							
Work full/part time or student	39%	37%	40%	36%	40%	41%	40%
Retired, disabled, unemployed	59%	61%	58%	62%	58%	57%	58%
Missing	2%	2%	2%	3%	2%	2%	2%
**Marital status**						
with partner	52%	53%	51%	59%	50%	53%	51%
without partner	44%	46%	44%	40%	46%	43%	46%
Missing	4%	2%	4%	1%	4%	4%	3%
**Family size more than 3**							
Yes	35%	25%	37%	15%	39%	20%	48%
Missing	6%	4%	6%	6%	6%	7%	5%
**Random or convenience sample**							
Random	82%	77%	83%	70%	85%	75%	87%
**Community**							
Near highway (≤500 m)							
Somerville	23%	33%	23%	27%	22%	31%	19%
Dorchester/South Boston	27%	35%	26%	27%	27%	23%	30%
Chinatown	24%	16%	24%	19%	25%	22%	24%
Urban background (≥1000 m)							
Somerville	6%	3%	6%	3%	7%	7%	7%
Dorchester/South Boston	9%	7%	9%	14%	8%	8%	10%
Malden	11%	5%	11%	10%	12%	10%	10%
**Time of residence in current address (years), mean (SD)**	14.2 (14.0)	21.0 (16.4)	13.9 (13.7)	17.9 (15.5)	14.4 (14.0)	21.1 (17.1)	12.8 (12.4)
**Estimated time since diagnosis, mean (SD)**	-	6.25 (5.33)	-	4.90 (4.61)	-	6.31 (5.42)	-
**Perceived Stress Scale, mean (SD)**	3.97 (3.24)	4.81 (3.75)	3.87 (3.17)	4.38 (3.30)	3.82 (3.20)	4.05 (3.21)	3.83 (3.23)
**log(Light to moderate exercise), mean (SD)**	1.66(1.03)	1.36 (1.12)	1.72 (1.00)	1.38 (1.10)	1.71 (1.01)	1.54 (1.06)	1.74 (0.97)

^1^ n = 57 for S/IHD was calculated from 86 participants who reported a diagnosis of S/IHD minus 29 who developed S/IHD prior to moving to their current address. ^2^ n = 73 for diabetes was calculated from 122 participants who reported a diagnosis or use of diabetes medication minus 51 who developed diabetes prior to moving to their current address. ^3^ n = 193 for hypertension was calculated from 320 participants who reported a diagnosis or use of hypertension medication minus 127 who developed hypertension prior to moving to their current address. ^4^ Body Mass Index (BMI) was calculated from 435 of the total population, with 29 IHD, 46 diabetes, and 125 hypertension cases.

**Table 2:** Distribution of time-activity adjusted annual average particle number concentrations (10^3^ particles·per·cm^3^) exposure by health outcomes.

TAA-PNC (particles/cm^3^)	Total	S/IHD *		Diabetes		Hypertension	
		Yes	No	Yes	No	Yes	No
	(n = 704)	(n = 57)	(n = 618)	(n = 73)	(n = 580)	(n = 193)	(n = 384)
Mean	21	22	21	20	21	21	20
SD	7.0	5.9	6.5	6.6	6.4	6.5	6.3
Min	9.0	10	8.8	8.8	9.0	9.3	8.8
25%	16	18	15	15	16	17	15
Median	22	22	21	22	22	22	21
75%	26	26	26	26	26	27	25
Max	35	33	35	33	35	34	35

* The participants who were diagnosed with each health outcome with an interval longer than the interval of moving to current address were excluded from the calculation of the corresponding health outcome.

**Table 3:** Mean TAA-PNC models for association with stroke/ischemic heart disease, diabetes, and hypertension.

Health Outcome	Age-Adjusted			Multi-Variable ^1^			Multi-Variable ^2^		
	OR	95% CI	*p*	OR	95% CI	*p*	OR	95% CI	*p*
S/IHD	1.14	(0.76, 1.72)	0.53	1.23	(0.79, 1.90)	0.36	1.35	(0.83, 2.22)	0.23
Diabetes	0.89	(0.61, 1.29)	0.53	0.78	(0.53, 1.15)	0.20	0.71	(0.46, 1.10)	0.12
Hypertension	1.19	(0.89, 1.59)	0.25	1.12	(0.82, 1.54)	0.46	1.14	(0.81, 1.62)	0.45

^1^ Adjusted for sex, education, and age. ^2^ S/IHD adjusted for sex, age, race, and PSS. Diabetes adjusted for age, education, race, random, and time of residence at current address. Hypertension adjusted for sex, age, education, race, random, time of residence at current address, and smoking status.

**Table 4:** Restriction analysis for participants with clinical measurement of BMI.

Health Outcome	Age-Adjusted			Multi-Variable ^1^			Multi-Variable ^2^		
	OR	95% CI	*p*	OR	95% CI	*p*	OR	95% CI	*p*
S/IHD	1.20	(0.72, 2.02)	0.48	1.61	(0.88, 2.92)	0.12			
Diabetes	1.22	(0.75, 1.97)	0.42	1.16	(0.67, 1.99)	0.60	1.09	(0.61, 1.96)	0.77
Hypertension	1.45	(1.00, 2.10)	0.05	1.50	(0.99, 2.29)	0.06	1.28	(0.81, 2.02)	0.30

^1^ Adjusted for sex, education, age, and BMI. ^2^ S/IHD not adjusted for more complex model due to limitation in sample size. Diabetes adjusted for age, education, BMI, time of residence at current address, and smoking status. Hypertension adjusted for age, education, BMI, race, time of residence at current address, and smoking status.

## Appendix A

### ***Study Design and*** ***Recruitment***

Details of the study design and methods are available in Fuller et al., 2013 [23]. We present a briefer description here.

The study utilized a three year, cross-sectional design in which we recruited and measured air pollution for one year in each of three neighborhoods in and near Boston, MA, USA (Figure 1). Community partners were involved in all aspects of the research, including design of the study and recruitment. We divided each study area into three strata based on distance to the highway: 0–50 m, 50–400 m and >1000 m. Addresses were obtained for all residences within the study areas and random samples were drawn. Recruitment was conducted in six languages: English, Spanish, Chinese (Mandarin and Cantonese), Vietnamese, Portuguese and Haitian Creole. The random sample was supplemented with convenience samples to obtain an optimal sample size. Up to five attempts were made at each address in the random samples.

Following informed consent, we administered orally a questionnaire to participants in their home. The questionnaire covered demographics (age, sex, race/ethnicity/national origin), SES (income and education), diagnosed illnesses and current medications (recorded from medication containers), health behaviors (smoking, physical activity, diet), social factors (perceived stress, recent life events), time spent in main microenvironments (home inside, home outside, at work or school, other and time on highways) for two recent days (a recent work/weekday and a recent weekend/non-work day) and recent exposure to combustion products (e.g., from cooking). We also asked about opening of windows, air conditioning and occupational exposures.

Following the in-home survey, participants were invited to attend up to two clinic sessions in or near their neighborhood. At the clinical sessions we drew blood samples, measured blood pressure (Model HEM711ACN2; Omron Healthcare, Kyoto, Japan), assessed blood lipid profile from a finger stick (Polymer Technology Systems; Indianapolis, IN, USA), and measured height and weight.

### ***Particle number concentration monitoring,*** ***regression modeling and exposure assessment***

PNC was measured near participant residences using the Tufts Mobile Air Pollution Laboratory (TAPL) [17] during the year in which participants filled out surveys and provided blood samples. In Somerville, Dorchester, Chinatown, and Malden, the TAPL was driven over the same route 2–6 times/day on 35–47 days and at different times between 04:00 and 22:00 on all days of the week. Monitoring was performed in all four seasons (~21–70 h each in spring, summer, winter, and fall). PNC was measured with a butanol condensation particle counter at 1-s intervals (CPC 3775, TSI, Shoreview, MN, USA).

Multivariate regression models of log-transformed hourly outdoor PNC were built, calibrated, and evaluated using the 1-s measurements and available covariate data [26,27]. Each model included both spatial (e.g., side of and distance to I-93, distance to nearest major road) and temporal (e.g., wind speed, wind direction, temperature, day of week, I-93 traffic volume and speed) variables to predict PNC across the study area (model R^2^: Somerville = 0.42; Dorchester/South Boston = 0.35; Chinatown = 0.23; Malden = 0.31). The adjusted-R^2^, root mean square error (RMSE), and prediction RMSE were stable under leave-one-day-out cross-validation [26]. Hourly ambient PNC were assigned to each of the 408 participant’s residence for every hour of the year (n = 8760 or 8784 h), using the coordinates of the residence in the appropriate neighborhood-specific model. Time-activity survey data for five micro-environments (inside home, outside home, at work, on highway, and other) were derived from the neighborhood-specific regression models for each participant. Details on the methods, demographic distribution, spatial patterns and repeatability can be found in Lane et al. 2013 [31]. Previously we compared residential ambient PNC and TAA-PNC exposure model performance in both an exposure misclassification [24] and health study framework [24,25]. We found an increase in the magnitude and strength of associations between TAA-PNC and inflammatory markers compared to residential ambient PNC. We have provided a summary of the hourly adjustments for each micro-environment.

- Inside home and outside home: Assuming 100% particle infiltration based upon previously published analyses of homes in our study population that showed a 0.95 median indoor-outdoor ratio (I/O) [32]; we assigned the residential ambient hourly average. We paired survey data on seasonal air conditioning (AC) with local temperature from weather stations and assumed that AC use occurred when ambient temperatures exceeded 21 °C (70 °F). We applied a PNC reduction of 25% or 28% (window and central AC, respectively) for infiltration during corresponding hours. AC reduction values were derived from a subset of CAFEH homes in which we conducted PNC indoor-to-outdoor sampling [32].
- Work Place: Participants who were employed full- or part-time in TRAP exposed occupations (e.g., bus drivers, crossing-guards, or parking ticket officers) were assigned an approximate higher exposure as the mean hourly residential PNC of all participants residing ≤50 m from I-93 for the hours they were at work. Participants without significant TRAP exposure (e.g., nurses, administrators, school teachers) were assigned a lower PNC exposure- i.e., the average hourly residential PNC of all participants residing in the urban background area (≥1000 m from I-93).
- Highway: We assigned PNC exposures for time spent on state and interstate highways as the on-highway concentration predicted by the Somerville model [27].
- Other: We assumed that urban background was the most likely exposure for any microenvironment in the metropolitan area, and assigned hourly residential average of all participants residing in the urban background.
